# The Role of Cellular Senescence in Obstructive Airway Diseases: From Mechanisms to Therapeutic Targets

**DOI:** 10.3390/ijms27083542

**Published:** 2026-04-16

**Authors:** Argyro Vrouvaki, Marina Moustaka Christodoulou, Georgios Hillas, Stelios Loukides, Evangelia Fouka

**Affiliations:** 2nd Respiratory Medicine Department, Medical School, National and Kapodistrian University of Athens, Attikon University Hospital, 12462 Athens, Greeceghillas70@yahoo.gr (G.H.); loukstel@med.uoa.gr (S.L.)

**Keywords:** cellular senescence, senescence-associated secretory phenotype (SASP), inflammation, senotherapeutics, COPD, asthma, bronchiectasis

## Abstract

Cellular senescence is a stress-induced type of irreversible cell cycle arrest, driven by telomere attrition, oxidative stress, DNA damage, mitochondrial dysfunction, oncogene activation, and chronic inflammation. Senescent cells remain metabolically active, secreting cytokines, chemokines, growth factors, matrix metalloproteinases, extracellular vesicles and oxidative mediators, comprising a senescence-associated secretory phenotype (SASP) that affects the tissue microenvironment. With aging, impaired immune clearance results in senescent cell accumulation, promoting inflammation, immunosuppression and fibrosis. Emerging evidence implicates cellular senescence in obstructive airway diseases, reflecting the lung’s continuous exposure to environmental and oxidative insults, and several pathways, including DNA damage response and p53/p21 and p16^INK4a^ signaling, telomere dysfunction, reactive oxygen species production, and mitochondrial defects, integrate stress signals to enforce senescence. In chronic obstructive pulmonary disease, a SASP-associated inflammatory milieu supports stress-induced tissue injury, while uncertainty still exists about the effects of chronic SASP on tumor suppression versus tumor promotion. In asthma, senescence processes have been associated with both Type(T)2-high and T2-low endotypes, underlying the interplay between environmental exposures, airway epithelial dysfunction and induced senescence mechanisms. Finally, in bronchiectasis, the neutrophilic, dysbiotic airway environment links dysregulated senescence with disease persistence and progression. Conventional therapies, antioxidants, serine protease inhibitors and novel senotherapeutic strategies represent promising approaches for therapeutic interventions.

## 1. Introduction

Cellular senescence is a response to cellular stress, characterized by a permanent arrest of cell cycle, during which cells cease to divide once they reach a critical telomere length instead of dying, a process known as replicative senescence [1,2]. This phenomenon becomes more prevalent with chronological aging, characterized by phenotypic, functional and metabolic cell reprogramming, resulting in altered morphology, dysregulated metabolism, and resistance to apoptosis [3]. However, in certain circumstances, cellular senescence can also be induced prematurely in response to diverse cellular stressors, including oxidative stress, DNA damage, and chronic inflammation, a condition referred to as stress-induced premature senescence (SIPS) [4,5]. Senescent cells release a plethora of bioactive modulators, such as cytokines, chemokines, growth factors, matrix metalloproteinases (MMP), oxidative molecules, and extracellular vesicles, collectively known as the senescence-associated secretory phenotype (SASP), that influence the surrounding tissue microenvironment [6].

Under physiological conditions, cellular senescence exerts beneficial effects in health, contributing to embryonic development, tissue homeostasis, and tumor suppression [7,8]. However, with aging, the accumulation and persistence of these non-dividing, stressed cells is associated with a shift towards a pro-inflammatory, pro-fibrotic mileu, which contributes to the pathogenesis of multiple age-related diseases, including neurodegenerative, cardiovascular and metabolic disorders, and cancer [9,10]. Consequently, targeting senescence cells or modulating the SASP has emerged as a promising approach for targeted intervention [11].

The lungs are particularly vulnerable to senescence-related processes, as a result of their constant exposure to environmental insults and reliance on effective epithelial repair mechanisms [12,13]. Accumulating evidence implicates cellular senescence in the pathogenesis of chronic obstructive airway diseases, such as chronic obstructive pulmonary disease (COPD) and asthma, in which accelerated lung aging and dysregulated SASP signaling have been reported [14,15]. However, a comprehensive understanding of the underlying mechanisms inducing cellular senescence in these diseases remains vague. In parallel, emerging data suggest a potential role for senescence in bronchiectasis, although relevant literature is limited and insufficient to preclude firm conclusions regarding its precise mechanistic and clinical impact [16].

In this narrative review we aim to present current evidence on the distinct and overlapping roles of cellular senescence in COPD, asthma and bronchiectasis, focusing on key pathophysiological drivers and exploring potential therapeutic implications and future research directions. To achieve this, we conducted a broad literature search across PubMed and Google Scholar databases, using the terms “cellular senescence” AND “COPD” OR “asthma” OR “bronchiectasis” AND “SASP”, including articles published between 1996 and 2026. Priority was given to international guidelines, large registry studies, randomized controlled trials and high-quality observational studies, with emphasis on their methodological robustness and clinical relevance.

## 2. Mechanisms of Cellular Senescence

Senescence constitutes an adaptive mechanism in response to cellular stressors that usually lead to genomic instability, such as telomere shortening, DNA damage, or oncogene activation [17]. In general, mild DNA damage results in cell cycle arrest, whereas more severe injury triggers cell death mechanisms such as apoptosis and autophagy [18]. The protein p53, often termed “the guardian of genome”, is essential for maintaining genomic stability and plays a critical role in determining cell cycle arrest [19,20]. This process is part of the DNA damage response (DDR), a phosphorylation-driven signaling event initiated by damage sensing, which is crucial for maintaining genome integrity and stability [21]. The DDR is regulated by damage-sensing modulators, including poly (ADP-ribose) polymerase (PARP), telangiectasia-muted (ATM) or ataxia telangiectasia and Rad3-related (ATR) kinases, as well as the p53/p21^cip1^ axis, which constitute crucial repair mechanisms of single-strand and double-strand DNA breaks [22]. This transduction of the damage signal to downstream DDR mediators and effectors finally exerts an appropriate repair response, according to the severity of DNA damage [23]. However, DNA damage is not the only mechanism triggering DDR; other stimuli, including telomere shortening, oncogene activation, oxidative stress and mitochondrial dysfunction, can independently activate it [24,25]. The molecular pathways through which these stressors activate the p53 axis and induce senescence are briefly outlined below.

### 2.1. Telomere Shortening—DNA Damage

Among cellular stressors, telomere shortening is one of the most well-described triggers of cellular senescence. Telomeres constitute repetitive DNA sequences at the ends of the chromosomes that progressively shorten after every cell division, serving to maintain the integrity of genetic material during cell replication [26]. Telomerase, the enzyme responsible for maintaining telomere length, is inactive in most somatic cells, with notable exceptions such as stem cells [27]. However, in normal somatic cells, telomeres eventually reach a critical length after a finite number of divisions, leading to structural compromise and activation of DDR [28]. Of note, telomerase is characteristically reactivated in approximately 85–90% of cancer cells, enabling them to maintain telomere length, bypass cellular senescence, and achieve replicative immortality [29].

Although telomere shortening is a hallmark of the senescence process, DNA damage in other parts of the genome can similarly induce this phenomenon. Several stressors, including genotoxic insults, such as ionizing or ultraviolet radiation, as well as pharmacological agents, like chemotherapeutics, may induce DNA lesions that compromise genomic integrity [30,31]. Both telomere erosion and persistent or irreparable DNA damage may activate DDR, ultimately leading to p53 pathway engagement, promoting senescence [32,33].

### 2.2. Oncogene Activation

Oncogene-induced senescence (OIS) is considered a tumor-suppressive mechanism, preventing the uncontrolled proliferation of cells with oncogenic mutations or DNA damage [34]. This process is initiated when oncogenes, such as *BRAF*, *AKT*, *E2F1* and *cyclin E*, become aberrantly activated or when onco-suppressor genes (i.e., *PTEN* and *NF1*) are inactivated [35]. The level of oncogene expression seems to play an important role for the induction of OIS, as evidence indicates that senescence driven by oncogenic Ras proteins was observed only when Ras signatures were overexpressed [36]. The tumor suppression protein p53 is activated indirectly in OIS through DDR, while mechanistic target of rapamycin (mTOR) kinase can directly phosphorylate p53, thereby enhancing its stability and activity [35]. This dual regulation of p53 ensures the implementation of a stable growth arrest, preventing the replication of oncogene-activated cells [22].

However, senescence can also exert oncogenic effects, as numerous SASP factors, including interleukin(IL)-8, IL-6, and vascular endothelial growth factor (VEGF), contribute to angiogenesis and epithelial cell proliferation, modulating the tumor microenvironment [37,38]. In breast and pancreatic cancer models, senescent fibroblasts developing within the tumor microenvironment suppress antitumor immunity through the SASP [39,40]. When these senescent fibroblasts are co-transplanted into immunodeficient mice, they were found to promote cancer cell proliferation and extracellular matrix (ECM) remodeling, likely by releasing MMPs [41]. Senescent stromal cells also inhibit the elimination of oncogenic Ras-expressing epithelial cells through cell competition [42]. Furthermore, senescent stromal cells are believed to promote cancer cell invasion and metastasis [43]. This dual role of senescence highlights its complex biological impact, emphasizing that although senescence acts as a defense mechanism to uncontrolled cell proliferation, the SASP can paradoxically contribute to tumor development by fostering a pro-tumorigenic environment.

### 2.3. Oxidative Stress

Oxidative stress is another major contributor to DNA damage and senescence. Reactive oxygen species (ROS), produced during normal cellular metabolism via enzymes such as Nicotinamide Adenine Dinucleotide Phosphate (NADPH) oxidase or induced by environmental stressors, can directly damage DNA through base oxidation and the formation of abasic sites and single- and double-strand DNA breaks [44]. These DNA lesions produced by ROS are particularly elusive at telomeric regions, where repair mechanisms are relatively inefficient. Consequently, oxidative stress may contribute to telomere dysfunction, even in the absence of critical telomere shortening, resulting in a sustained DDR [45]. This pathway highlights the critical role of oxidative stress in both aging and in age-related diseases and cancer, in which telomere maintenance and DNA repair mechanisms are often compromised [33].

### 2.4. Mitochondrial Dysfunction

The role of mitochondria in the senescence process is dual, serving both as sources and targets of oxidative stress. Robust evidence suggests that ROS-induced mitochondrial DNA mutations are well-associated with mitochondrial dysfunction and premature aging [46]. In the context of senescence, mitochondria undergo structural changes that are typically associated with significant increases in size and volume [47]. These increases result mainly from accumulation of dysfunctional organelles due to defective mitophagy degradation process [48]. In the lungs, cigarette smoke induces senescence by inhibiting mitophagy in fibroblasts and small airway epithelial cells [49], whereas activation of mitophagy, particularly via inhibition of the mTOR pathway, postpones senescence onset [50,51]. This phenomenon is often accompanied by a unique type of SASP lacking the increased expression of the pro-inflammatory cytokines IL-6 and IL-8 [52]. Impaired mitochondrial dynamics and bioenergetics, characterized by reduced production of Adenosine Triphosphate (ATP) and imbalances in the Adenosine Monophosphate (AMP):ATP and Adenosine Diphosphate (ADP):ATP ratios, can ultimately promote cell cycle arrest [53].

However, beyond metabolic failure, mitochondrial dysfunction can directly promote inflammation through activation of damage-associated molecular patterns (DAMPs), inflammasomes and immune cells, contributing to the pathogenesis of a wide range of disorders, such as cancers, cardiovascular, metabolic and neurodegenerative diseases, sepsis and autoimmunity [54]. Mitochondrial dysfunction is consistently found across multiple senescence contexts, including replicative, stress-induced, oncogene-induced and telomere-associated senescence [55,56]. Notably, selective removal of dysfunctional mitochondria reduces SASP, while, in contrast, complete mitochondrial depletion promotes senescence persistence, highlighting their dual role [57].

### 2.5. Immunosenescence

Senescent cells, despite their growth arrest, remain metabolically active and significantly affect their the local microenvironment through the secretion of SASP mediators, modulating the behavior of both senescent and surrounding non-senescent cells in an autocrine and paracrine manner, respectively [6].

However, immune cells age as well, through a process known as immunosenescence, which results in compromised clearance capacity, and the subsequent accumulation of senescent cells [58]. Consequently, an amplifying mechanism is created, where structural cells express the senescent phenotype and aged immune cells fail to eliminate them. A significant consequence of sustained accumulation of SASP factors with age is the establishment of a chronic, low-grade inflammatory state termed inflammaging, characterized by persistent activation of proinflammatory cytokine pathways [59,60]. This increased expression of SASP modulators and the resulting inflammaging are closely associated with compensatory immunosuppression, mediated by the recruitment of T regulatory (Tregs) cell recruitment and the production of tumor-growing factor (TGF-β) and IL-10, both promoting wound healing and tissue regeneration and inducing fibrosis [61].

This phenomenon creates a vicious cycle among immunosenescence, inflammaging and immunosuppression, which is prominently implicated in various age-related diseases [62]. However, SASP also plays a beneficial role in the maintenance of tissue integrity, assisting the clearance of senescent cells through the recruitment of innate immune cells, such as neutrophils, macrophages, cytotoxic Natural-Killer (NK) cells and NK-T lymphocytes [63]. Understanding this dual role of SASP, in both driving chronic inflammation and supporting immune surveillance, is crucial for the development of therapeutic strategies targeting senescence in aging and disease.

## 3. Senescence in Obstructive Airway Diseases

Chronic airway diseases, such as COPD, asthma, and bronchiectasis are characterized by persistent inflammation that drives tissue damage and functional decline [64]. This chronic inflammatory environment promotes the release of pro-inflammatory cytokines, oxidative stress and DNA damage, accelerating premature senescence of airway cells, independently of chronological aging [4,65]. The relative contribution of major senescence-inducing mechanisms across COPD, asthma, and bronchiectasis is summarized in Table 1.

### 3.1. COPD

The concept that COPD is a disease characterized by accelerated lung aging has been extensively investigated, as the aging lung shares a plethora of characteristics with the concept of “COPD lung” [66,67]. The molecular and structural changes observed in normal aging appear to be amplified and occur prematurely in individuals with COPD, with the most prominent being airspace enlargement due to excessive oxidative stress, and chronic inflammation [68,69].

#### 3.1.1. Inflammatory Microenvironment and Stress-Induced Senescence in COPD

Senescence markers in the COPD lung tissue have been extensively investigated, with the identification of cytoplasmic, nuclear, and SASP-associated molecules establishing a strong association between the two entities [70,71]. The inflammatory milieu characteristic of COPD closely resembles that of SASP, as alveolar macrophages secrete common SASP components [i.e., IL-6, IL-8, and tumor necrosis factor alpha (TNF-α)], which perpetuate neutrophilic inflammation and tissue remodeling, providing a prototypical microenvironment for the induction of SIPS and perpetuating the cycle of inflammation and cellular stress within the lungs [72,73]. Exposure to SASP cytokines constitutes a persistent oxidative and inflammatory stressor, contributing to a sustained chronic inflammatory response that affects adjacent cells in a paracrine manner, establishing a feed-forward loop that perpetuates inflammation [74,75].

Preclinical studies have provided compelling evidence suggesting involvement of cellular senescence in the pathobiology of COPD. Plasminogen activator inhibitor-1 (PAI-1), a key component of SASP, has been found increased in sputum samples of COPD patients, suggesting a potential role of cellular senescence as a contributor to the chronic inflammation seen in that disorder [76]. In addition, endothelial colony-forming cells and epithelial cells derived from COPD patients in an ex vivo model demonstrate increased expression of common senescence markers, such as senescence-associated β-galactosidase (SA-β-gal), p16, and p21, in comparison to control subjects [77,78]. Mechanistic evidence further supports a link between COPD pathobiology and senescence. A recently published study suggests that increased interferon signaling in epithelial cells of COPD patients (ex vivo model) may promote and sustain senescence, through activation of DDR–associated pathways, including cyclic GMP-AMP synthase (cGAS)– stimulator of interferon genes (STING) and downstream Janus kinase/signal transducer and activator of transcription (JAK–STAT) signaling [79]. This SASP-like inflammatory signaling is believed to exacerbate tissue damage and remodeling in COPD; however, we should acknowledge that these markers are not senescence-specific and may also reflect broader cellular stress and inflammation [80].

#### 3.1.2. The Onco-Suppressive Role of Senescence in COPD

The frequent concurrence of COPD and lung cancer suggests shared pathogenic pathways, prompting interest in intrinsic mechanisms that may constrain malignant transformation in chronically injured lung tissue [81]. In this context, cellular senescence emerges as a critical tumor-suppressive barrier, which imposes irreversible cell-cycle arrest in damaged or stressed cells, limiting the early stages of carcinogenesis [82]. In contrast, the ongoing accumulation of senescent cells can impair tissue regeneration, establishing a pro-inflammatory setting that may eventually favor tumor development by altering the local microenvironment milieu [83]. This dual role of senescence, both suppressing and, under certain conditions, promoting tumor development, creates a complex paradox regarding the overall impact of senescence on lung cancer risk in patients with COPD.

### 3.2. Asthma

Asthma is a heterogeneous chronic inflammatory airway disease, with current classification emphasizing mechanistic endotypes rather than clinical phenotypes to better reflect underlying immune and inflammatory pathways [84]. In this context, asthma endotypes are broadly classified as T2-high, typically eosinophilic, and T2-low, often neutrophilic, that differ in their underlying mechanisms and responses to environmental exposures, aging and inflammatory stress [85].

Recent research has highlighted cellular senescence as a potential contributor to asthma pathogenesis [14]. Senescent cells accumulate in the lungs with age, in response to chronic inflammation and other stressors including allergens, pollutants and infections, modulating the tissue microenviroment and contributing to asthma pathogenesis [86].

#### 3.2.1. Inflammation–Senescence Crosstalk in Asthma

Asthma pathology is driven by a complex and dynamic inflammatory milieu shaped by both innate and adaptive immune responses, which closely interact with cellular senescence to amplify airway dysfunction and remodeling [87]. Environmental exposures, including air pollutants, allergens and cigarette smoke, act as chronic stressors that promote mitochondrial dysfunction, oxidative stress and DNA damage, and may trigger stress-induced senescence within the asthmatic airway [13]. These processes appear to contribute to the activation of key senescence pathways, such as the p53/p21 and p16^INK4a^ axes, and facilitate the adoption of a SASP by senescent airway structural cells and immune cells, characterized by the release of IL-6, IL-8 and matrix-modifying factors, which may further perpetuate airway inflammation, compromise epithelial barrier integrity and impair tissue repair, thereby driving airway remodeling [88,89,90].

Consistent with this paradigm, experimental evidence from a *murine* model has demonstrated that cellular senescence induced by diesel exhaust particles enhances allergen sensitization and promotes the development of *murine* asthma, characterized by increased airway responsiveness and eosinophilic airway inflammation [91]. In addition, a recent study employing computational analysis of *human*-derived transcriptomic datasets found that alveolar macrophages in asthma exhibit pronounced senescence-associated gene signatures, linked to increased cytokine production and sustained airway inflammation. Interestingly, activation of peroxisome proliferator-activated receptor-γ (PPARγ), a key regulator of oxidative-reduction and cytokine-related genes, attenuated both the senescent signature and cytokine output of these macrophages, suggesting senescence-associated inflammatory amplification may be, at least in part, therapeutically reversible [92]. Collectively, these findings suggest that cellular senescence acts as a mechanistic link between aging, environmental stressors, and chronic airway inflammation in asthma, with important implications for disease susceptibility, progression, and structural airway changes.

#### 3.2.2. Cellular Senescence Across Asthma Phenotypes and Endotypes

Cellular senescence is increasingly recognized as a biologically relevant process in asthma pathogenesis and progression [14,93]. Given that asthma endotypes are driven by distinct immune pathways, it is debated whether senescence manifests differently in T2-high and T2-low disease, while remaining mechanistically relevant to both.

Ιn T2-high asthma, oxidative stress and sustained exposure to T2-associated cytokines, such as IL-4, IL-5 and IL-13, may drive premature senescence in airway epithelial and immune cells, particularly in older adults or those with severe disease [94]. Immune alterations related to aging, such as reduced phagocytic function, increased production of pro-inflammatory mediators, as well as the emergence of SASP, may further accelerate this process, creating a local microenvironment that favors chronic inflammation, mucus hypersecretion and structural remodeling [95].

By contrast, T2-low asthma is associated with T helper (Th) 1 and Th17 immune responses, characterized by enhanced expression of toll-like receptors, including TLR2 and TLR4 in airway epithelial cells, and increased release of pro-inflammatory cytokines, such as interferon (IFN)-γ, TNF-α, IL-8, IL-17A, IL-17F and IL-22 [96,97]. Increased expression of senescence-related genes, including *ETS2*, *ETS1* and *AURKA*, has been reported in sputum samples from patients with severe asthma compared with healthy controls, supporting the link between non-T2 inflammation and senescence and suggesting a role in disease severity and persistence [86]. Notably, upregulation of senescence-associated pathways, including the p53/p21 and p16^INK4a^ axes, has been demonstrated ex vivo in airway smooth muscle cells from elderly asthmatics. These cells also exhibited higher expression of other SASP mediators, such as IL-6, IL-8 and fibronectin, compared with age-matched non-asthmatic controls, a finding associated with airway wall thickening, subepithelial fibrosis, and airway remodeling [89]. Collectively, these observations suggest that aging promotes a state of chronic, low-grade inflammation and increased of senescent cell burden, contributing to increased disease severity, fixed airflow limitation and treatment refractoriness [95].

#### 3.2.3. Cellular Senescence and Lung Cancer Risk in Asthma

Asthma has long been investigated as a potential contributor to lung cancer risk, with an early meta-analysis reporting a significant association between asthma and lung cancer incidence [98]. This hypothesis is further supported by a more recent Mendelian randomization analysis suggesting a potential causal relationship, in line with prior observational studies [99].

From an oncosuppressive perspective, cellular senescence in asthma constitutes a fundamental intrinsic mechanism that limits the proliferative capacity of damaged or stressed cells, enforcing stable cell-cycle arrest and constraining the expansion of genomically unstable cells [93]. However, direct mechanistic evidence linking senescence burden or SASP in asthma to lung carcinogenesis is limited and less clearly defined than in COPD, in which cumulative smoking-related injury is a key driver of carcinogenic risk [100]. Defining the conditions under which senescence retains its tumor-suppressive capacity or becomes maladaptive will be critical for understanding long-term oncologic outcomes in asthma.

### 3.3. Association of Cellular Senescence with Bronchiectasis

Bronchiectasis is a chronic, heterogeneous airway disease defined by permanent bronchial dilation and clinically characterized by chronic cough, sputum production and recurrent infections [101]. Bronchiectasis features predominantly neutrophil-driven inflammation, marked by excessive neutrophil activity, delayed apoptosis, protease release and neutrophil extracellular traps (NET) formation, all of which contribute to tissue injury, while other immune cells, including macrophages, B- and CD4^+^ T lymphocytes, further shape inflammation and impact disease progression [102,103]. Microbial dysbiosis, mainly associated with Pseudomonas- and Haemophilus-dominated microbiome profiles, promotes recurrent cycles of chronic infection and inflammation and has been shown to be linked to severe disease and frequent exacerbations [104].

These features create a setting in which repeated epithelial injury, increased protease activity and immune activation can converge on pathways of cellular aging and dysfunction [105]. Despite the scarcity of mechanistic studies establishing causality between bronchiectasis and cellular senescence, preliminary observations suggest a possible link. An early *human*-tissue study has shown that epithelial cells from patients with severe bronchiectasis exhibit shortened telomeres, increased expression of p21 and decreased levels of the NAD+ dependent histone deacetylase sirtuin 1 (SIRT1) compared to healthy age-matched controls, findings consistent with higher senescence burden [16]. Similarly, a recent cross-sectional study using indirect biomarker-based inference reported that patients with bronchiectasis demonstrated significantly lower serum SIRT1 levels and higher MMP-9 concentrations compared to healthy controls [106]. Additionally, senescence appears to be implicated in cystic fibrosis-related bronchiectasis as well. Chronic infection, oxidative stress and chronic, mainly neutrophilic, inflammation appear to activate p16- and p21-mediated pathways and intensify SASP mediated inflammation, as well as impaired airway tissue repair [107,108,109,110]. Although these processes may worsen airway damage and bronchiectasis progression, robust data linking cystic fibrosis transmembrane conductance regulator (CFTR) dysfunction to senescence is lacking and further research is warranted [111]. Collectively, these findings suggest that senescence-related alterations and the inflammatory milieu may interact as interrelated components of bronchiectasis pathophysiology.

## 4. Future Directions

Cellular senescence is increasingly recognized as a disease-relevant process, rather than merely a part of natural aging. Chronic airway diseases such as COPD, asthma and bronchiectasis share inflammatory features that often overlap with senescence characteristics, with airway epithelial cells of patients with these conditions consistently exhibiting increased levels of SASP [112]. However, significant gaps remain in understanding whether senescence develops prematurely and to a greater extent in these disorders, or if its functional consequences differ compared to age-matched controls [63,67,89]. Therefore, future research should prioritize longitudinal, age-matched and more clinically oriented studies to clarify whether senescence contributes to disease pathology or is only a bystander epiphenomenon.

Another key challenge lies in the identification of senescence as a reliable biomarker in human lung tissue. Although multiple markers of cell cycle arrest, such as nuclear inhibitors p16^INK4a^ and p21^CIP1^, cytoplasmic proteins, such as SA-β-gal and lipofuscin, as well as SASP components (i.e., IL-1a, IL-6, and IL-8, and MMPs), are available via immunochemistry, no single marker is sufficiently specific or stable over time [113,114,115,116]. Combined approaches are therefore required, particularly in tissue samples where cellular stress and inflammation may complicate interpretation.

Beyond detection, a critical future direction is the development of targeted therapies aimed at selectively combating the effects of aging in a swiftly evolving field, referred to as senotherapeutics. Agents designed to selectively remove senescent cells (known as senolytics) and agents targeting compounds of SASP (referred to as senomorphics) are set to offer protection against a broad range of serious pathological conditions and age-related disorders [117]. Potential senolytic and senomorphic effects of established treatments for chronic airway disease are under thorough investigation. Azithromycin has been shown to modulate inflammatory responses and promote the clearance of senescent cells in asthmatic lungs, while inhaled corticosteroids have been demonstrated to protect against endothelial senescence in COPD patients [77,118].

As research progresses, drugs commonly used in other pathologies are found to have senolytic effects in aging lungs. Metformin, a classic antidiabetic drug, combats airway inflammation and remodeling in asthmatic murine models, by targeting senescence pathways nuclear factor kappa B (NF-Κβ) and AMP-activated protein kinase (AMPKα) [119,120]. Similarly, vitamin D, a molecule not traditionally used in treating obstructive conditions, improves recovery from asthma-induced lung injury in rats by regulating hypoxia-inducible factor 1-alpha (HIF-1α)/Notch1 signaling during autophagy [121].

Furthermore, as oxidative stress is a known trigger of SIPS, trying to prevent senescence using antioxidants shows promise. Synthetic antioxidants, such as N-acetylcysteine, and natural compounds, like vitamin C, have shown efficacy in attenuating oxidative stress and senescence markers [122]. Similarly, novel agents such as NADPH oxidase inhibitors, that limit ROS production, and sirtuin-activating agents, which are synthetic analogs of reservatrol, an antioxidant believed to activate anti-aging factor SIRT1, have been shown to reduce senescence in epithelial progenitor cells from COPD patients [123,124].

In this context, targeting neutrophil-mediated injury may help interrupt the cycle of inflammation, senescence and structural damage. Brensocatib, a reversible inhibitor of dipeptidyl peptidase-1 (DPP-1), an enzyme required for the maturation of neutrophil serine proteases (NSP), has been found to reduce NSP activity in patients with bronchiectasis, with favorable clinical outcomes across all key baseline disease characteristics [125,126].

By contrast, senotherapeutics, although promising, are far from clinical application, since most data that is currently available is based on animal or cellular models [119,121]. Novel senolytic agents, modulators of nuclear factor erythroid 2-related factor 2 (Nrf2) acetylation, and interventions targeting senescence-related genes have shown favorable potential to modify the airway microenvironment and reduce structural remodeling in experimental models [127,128,129]. Studies on senotherapeutics in the context of COPD and bronchiectasis are still limited, focusing on agents commonly used in standard treatments, such as azithromycin [130,131]. More clinical trials are needed to fully comprehend the benefits of such treatments, as well as the possible implications rising from senescent cell elimination.

Other potential drugs targeting senescence in chronic airway disease and the proposed mechanism of action are outlined in Table 2.

## 5. Conclusions

**Figure 1:**
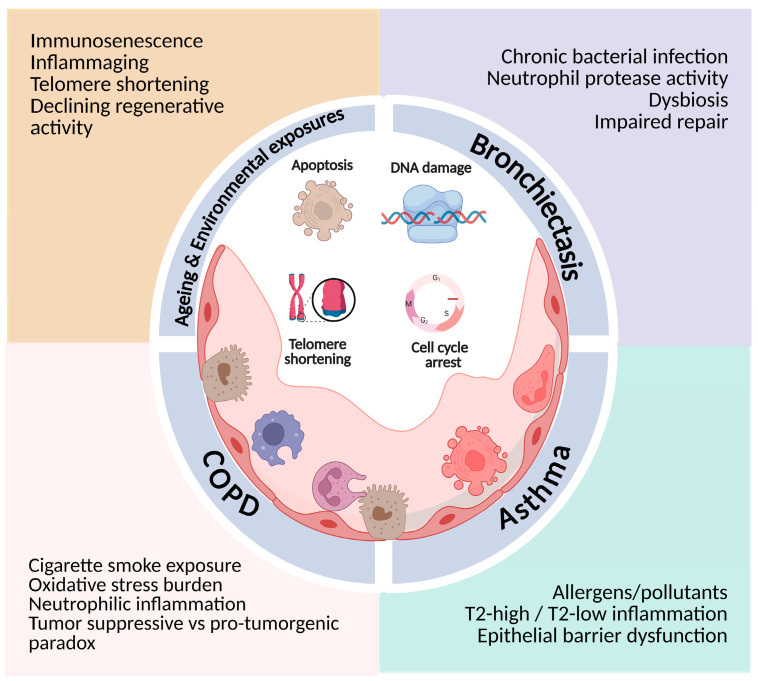
Cellular senescence as a shared pathobiological pathway across chronic airway diseases. The figure illustrates cellular senescence as a convergent mechanism linking aging and disease-specific insults across chronic airway diseases (COPD, asthma, bronchiectasis). Environmental and inflammatory stressors induce DNA damage and telomere dysfunction, leading to irreversible cell-cycle arrest, impaired epithelial repair and persistent inflammation. These senescence mechanisms are shared by all conditions but are differently shaped by disease-specific microenvironments: cigarette smoke and oxidative stress–driven inflammation in COPD; allergen and pollutant exposure, as well as endotype-specific (T2-high and T2-low) inflammation in asthma; and chronic infection, microbial dysbiosis and neutrophil protease activity in bronchiectasis. Collectively, these contexts promote airway damage, airway remodeling and disease progression. Created in BioRender. Fouka, E. https://BioRender.com/94cdx4x (accessed on 22 February 2026).

Cellular senescence constitutes a key biologic phenomenon linking genomic instability, oxidative stress, and mitochondrial dysfunction to chronic inflammation, tissue damage and remodeling, and increased cancer risk in obstructive airway diseases. The available evidence suggests that cellular senescence in chronic airway diseases is not merely a feature of premature aging, but also a mechanistically relevant factor contributing to disease chronicity and structural damage. Age-related processes further amplify these effects, while each disease microenvironment modulates downstream outcomes, collectively promoting airway damage, remodeling and disease progression as seen in Figure 1. More importantly, cellular senescence in chronic airway diseases should be regarded as a significant disease amplifier, with its effects depending on host aging, environmental burden, and inflammatory endotypes.

## Figures and Tables

**Table 1:** Relative contribution of major senescence-inducing mechanisms across chronic airway diseases.

Senescence Driver	COPD	Asthma	Bronchiectasis
Telomere shortening	*++*	*+*	*++*
DNA damage (DDR)	*+++*	*++*	*+*
Oncogene activation	*+*	*+*	*−*
Oxidative stress	*+++*	*++*	*++*
Mitochondrial dysfunction	*+++*	*++*	*+*
Chronic inflammation/SASP	*+++*	*+++*	*+++*

Semi-quantitative grading reflects the relative strength of evidence and mechanistic prominence based on currently available literature. +++: dominant driver; ++: important contributor; +: moderate/conditional role; −: limited or no evidence. DDR: DNA Damage Response; SASP: senescence-associated secretory phenotype; COPD: chronic obstructive airway disease.

**Table 2:** Pharmacologic strategies with potential senomorphic or senolytic activity in chronic airway diseases.

Category	Molecule	Condition Studied	Proposed Senescence-Related Mechanism	Clinical Status	Drug Category
Repurposed agents (senomorphic)	Azithromycin	Asthma, COPD, bronchiectasis	Modulates SASP signaling; attenuates PI3K/Akt/mTOR pathway; promotes clearance of senescent cells; reduces FOXO3A/CCND1-mediated senescence	Approved macrolide; used long-term in selected phenotypes [118,130,131]	Human cells, in vitro
	Inhaled corticosteroids (ICS)	COPD	Protect against endothelial progenitor cell senescence; reduce inflammatory signaling	Standard COPD therapy [77]	Human cells, ex vivo
	Metformin	Experimental asthma models	Activates AMPKα; inhibits NF-κB; restores autophagic flux; reduces oxidative stress-induced senescence	Approved antidiabetic; investigational in airway disease [119,120]	Murine model
	Vitamin D	Experimental asthma models	Modulates HIF-1α/Notch1 signaling; regulates autophagy-associated pathways; attenuates senescence	Supplement; investigational role [121]	Murine model
Antioxidant/redox modulators	N-Acetyl L-Cysteine	COPD (experimental)	ROS scavenging; attenuates oxidative stress-induced premature senescence (SIPS)	Approved mucolytic [122,132]	Murine model
	Vitamin C	Experimental	Antioxidant; reduces oxidative damage and senescence markers	Supplement [122]	Murine models
	NADPH oxidase inhibitors	COPD (preclinical)	Reduce ROS production; mitigate oxidative DNA damage and cellular senescence	Experimental [124,133]	Murine models
	Sirtuin-activating compounds (SIRT1 activators)	COPD (preclinical)	Restore SIRT1 activity; improve mitochondrial function; suppress senescence pathways	Experimental [123,124]	Human cells, ex vivo
Targeted pathway inhibitors	Brensocatib (DPP-1 inhibitor)	Bronchiectasis	Reduces neutrophil serine protease activation; limits inflammation-driven tissue damage and secondary senescence	Phase 3 positive; regulatory evaluation ongoing [125,126]	Human trials, in vivo
Emerging/experimental senotherapeutics	Nrf2 acetylation modulators	Experimental asthma	Reduce oxidative stress-driven senescence; attenuate airway remodeling	Preclinical [127]	Murine model

The agents included present senotherapeutic potential and are not established senotherapeutic molecules. COPD: chronic obstructive airway disease; SASP: senescence-associated secretory phenotype; PI3K: Phosphoinositide 3-kinase; mTOR: Mechanistic target of rapamycin; FOXO3A: Forkhead Box O3; CCND1: Cyclin D1; ICS: Inhaled corticosteroids; AMPKα: AMP-activated protein kinase; NF-κB: Nuclear factor kappa beta; HIF-1α: Hypoxia-Inducible Factor 1-alpha; ROS: Reactive oxygen species; SIPS: stress-induced premature senescence; NADPH: Nicotinamide adenine dinucleotide phosphate; SIRT1: Sirtuin-1; DPP-1: dipeptidyl peptidase I; Nrf2: Nuclear factor erythroid 2-related factor 2.

## Data Availability

No new data were created or analyzed in this study. Data sharing is not applicable to this article.
